# Comparison of the prevalence of major transfusion-transmitted infections among Iranian blood donors using confidential unit exclusion in an Iranian population: Transfusion-transmitted infections among Iranian blood donors

**Published:** 2011-01-01

**Authors:** Azadeh OmidKhoda, Ahmad Gharehbaghian, Mostafa Jamali, Naser AhmadBeigi, Seyed Mahmoud Hashemi, Abas Rahimi, Masoud Soleimani

**Affiliations:** 1 Iranian Blood Transfusion Organization (IBTO) Research Center, Tehran, IR Iran; 2 Department of Hematology, Faculty of Medicine, Tarbiat Modares University, Tehran, IR Iran; 3 Department of Stem Cell, Stem Cell Technology Co. Ltd, Tehran, IR Iran; 4 Department of Immunology, Faculty of Medicine, Tarbiat Modares University, Tehran, IR Iran; 5 Department of Statistic, Faculty of Medicine, Tehran University, Tehran, IR Iran

**Keywords:** Confidential unit, Exclusion, Risk factors, Iranian, Blood donors

## Abstract

**Background:**

Nucleic acid amplification testing is recommended for screening blood donations; however, they are not widely available in developing countries such as Iranian. Confidential unit exclusion (CUE) gives blood donors the opportunity to confidentially indicate whether their blood is or is not suitable for transfusion to others. Hoewever, its effectiveness in improving blood safety has recently been questioned by the blood banking community.

**Objectives:**

The purpose of this study was to determine the efficacy of CUE in Iran.

**Patients and Methods:**

Data on transfusion-transmitted disease markers (HBs Ag, HCV Ab, HIV Ab, RPR) were extracted from a database of voluntary blood donations in 2006 at the Tehran Blood Transfusion Center. The prevalence of markers were compared between CUE-positive ("should not use") and CUE-negative ("can be used") donations.

**Results:**

CUE-positive donations had significantly higher risk of HBV and HCV markers (odds ratio (95% confidence interval)7.5 (5.4-10.5) and 5.3 (2.5-11.3), respectively). No HIV or syphilis markers were detected in either group.

**Conclusions:**

CUE is an effective option for identifying donors with increased risk of HBV and HCV markers.

## Background

The blood supply today is safer than ever, mainly due to more careful donor selection and, in developed countries, the use of nucleic acid amplification testing (NAT) for transfusion-transmitted diseases (TTDs) such as HBV, HCV, HIV and syphilis. A highly effective donor selection program including a confidential unit exclusion (CUE) option and donor medical histories has special importance in developing countries such as Iran, where NAT has not been implemented and approximately 60% of participants are first-time donors, for whom the prevalence of positive serological tests for TTDs is higher than in repeat donors [1]. In the United States, the use of CUE was recommended by the US Food and Drug Administration in 1986 [2]. CUE gives a donor the opportunity to indicate in confidence that the donated unit should not be used for transfusion. Donors who choose CUE may know or suspect that they have risk factors, and may donate so as to obtain test results for infections such as HBV, HCV and HIV.

A number of studies have examined CUE effectiveness. Pindyck et al. found significant differences in the prevalence of positive laboratory tests in donors excluding themselves compared to donors who did not exclude themselves [3]. A separate study reported that donors who excluded themselves were more likely to be HIV Ab positive than donors who did not exclude themselves. However, infrequency of confidential exclusion by window-period donors causes the CUE option to have minimal impact on blood safety [4]. In another study, low sensitivity and low positive predictive value was seen in a CUE implementation [5]. CUE has been used at the Tehran Blood Transfusion Center (TBTC) since 2003. However, no study has examined CUE effectiveness in Iran.

## Objectives

This study evaluated the efficacy of CUE for blood safety by comparing the prevalence of TTD markers in CUE users and non-CUE users at TBTC.

## Patients and Methods

Our analysis is based upon a database representing all donations at TBTC. TBTC receives voluntary blood from 25 collection centers across the city. Each center uses a standardized blood donor record. This record contains demographic information, donor status (first-time or repeat), medical history, physical examination, CUE designation, blood group, TTD markers, and confirmatory testing results for positive donations. This information for each donation is added to the TBTC database.In the Iranian Blood Transfusion Organization (IBTO), in order to ensure blood safety, all donors must undergo a complete medical history and fill out the CUE form. Each donor's medical history is reviewed by a qualified physician through a questionnaire and a personal interview. The vast majority of items in the questionnaire are intended to determine if the donor has risk factors for exposure to an increasing number of proven or potential TTDs. The physician rejects those donors with any risk factors for TTDs, such as intravenous drug abuse, history of blood transfusion, tattoo, acupuncture, body piercing, surgery, allogeneic and syngeneic transplantation, tooth extraction, liver disease, jaundice, lymphadenopathy, in addition to those with positive serologic markers for TTDs.

After undergoing a medical history, the CUE form is given to the donor. This form contains information about TTDs and has two options, as follows:

"My blood can be used for patients."

"My blood should not be used for patients."

The donor must choose one of the two options by ticking a check mark and then place this form into the certain box in the center. If the donor chooses the should-not-be-used option (CUE positive), the unit and all products from that unit will be destroyed, regardless of its test results.In this study, data from voluntary first-time and repeat donations in 2006 were analyzed. The data were divided into two groups: CUE positive and CUE negative. As the population of CUE-negative donations was very large compared to the population of CUE-positive donations, a random sample of CUE-negative donations that was four times the size of the CUE-positive population was taken. Descriptive statistics were calculated for both groups. The prevalence rates of HBV, HCV, HIV and syphilis markers among first-time and repeat donors in both groups were then calculated. For each marker, the prevalence was calculated as the number of confirmed-positive donations divided by the total number of donations [6]. The following screening tests for TTDs were used: HBs Ag (Enzygnost HBs Ag 5.0, Dade Behring), anti-HCV (Hepanostika microelisa system, bioMérieux, Marcy l'Etoile, France), HIV Ag/Ab (Vironostika, HIV Uni-Form II Ag/Ab microelisa system, bioMérieux) and RPR (Enison, RPR slide test). Confirmatory tests were performed on all repeatedly reactive donations using the following tests: HBs Ag (HBs Ag Confirmatory Assay, Dade Behring), anti-HCV (HCV RIBA 3.0, Genelabs Diagnostics), Anti-HIV (HIV BLOT 2.2, Genelabs Diagnostics) and RPR (FTA-ABS Test System, Mardx Diagnostics). A first-time donor was defined as an individual who never donated before; a lapsed donor was defined as an individual whose previous donation was more than 1 year ago; and a repeat donor was defined as an individual whose previous donation was less than 1 year ago. Repeat donors with CUE were those who used the CUE option in their last donation attempt.

### Statistical analysis

Statistical analysis was carried out using SPSS 16 software and comparisons were evaluated with chi-square test. A p<0.05 and a 95% confidence interval (CI) of odds ratio that does not contain 1 was considered significant.

## Results

### Descriptive analyses in CUE-positive versus CUE-negative donors

There were 2,864 (0.92%) CUE-positive and 307,782 (99.08%) CUE-negative donations. In order to compare these two groups, 11,456 CUE-negative donations were chosen randomly. Table 1 shows descriptive statistics for CUE-positive and CUE-negative donors. In both groups, the majority were first-time donors and the large majority were male. Although the number of the first-time donors was higher than repeat donors in both groups, there was a significant relationship between first-time donation and being CUE positive (p<0.0001, odds ratio: 2.14, 95% CI: 1.94-2.35). There was also a significant correlation between male gender and CUE-positive donations ( p<0.0001, odds ratio: 2.35, 95% CI: 1.9-3).

**Table 1:** Descriptive statistics of CUE-positive and CUE-negative donors in 2006

**CUE**	**Number of donation**	**Male **(% population subset)	**Female **(% population subset)	**First time **(% population subset)	**Repeat **(% population subset)	**Lapsed **(% population subset)
**Negative**	11456	10660 (93)	796 (7)	6093 (53.1)	4297 (37.5)	1066 (9.3)
**Positive**	2864	2776 (97)	88 (3)	1999 (69.8)	658 (23)	207 (7.2)

### Prevalence of TTD markers in CUE-positive versus CUE-negative donors

Table 2 compares the prevalence rates of TTD markers between CUE-positive and CUE-negative donations. The prevalence of confirmed HBs Ag was 3.4% (99/2,864) among CUE-positive donations and 0.5% (54/11,456) among CUE-negative donations (p<0.0001, odds ratio: 7.5, 95% CI: 5.4-10.5). The prevalence of confirmed anti-HCV was 0.5% (54/11,456) among CUE-positive donations and 0.1% (12/11,456) among CUE-negative donations (p<0.0001, odds ratio: 5.3, 95% CI: 2.5-11.3). No anti-HIV or syphilis was detected.

**Table 2:** Prevalence of TTD markers in CUE-positive versus CUE-negative donors in 2006

**Marker**	**CUE**	**Total number of donations**	**Number positive for marker**	**Prevalence **(%)	**p-value**	**Odds ratio**(95% CI)
HBs Ag	Negative	11,456	54	0.5	<0.0001	7.5(5.4-10.5)
	Positive	2,864	99	3.4		
HIV	Negative	11,456	0	0	0.3	3.9(0.2-64)
	Positive	2,864	0	0		
HCV	Negative	11,456	12	0.1	<0.0001	5.3(2.5-11.3)
	Positive	2,864	16	0.5		
Syphilis	Negative	11,456	0	0	0.3	3.9(0.2-64)
	Positive	2,864	0	0		

## Discussion

This study assessed the usefulness of CUE by comparing the prevalence rates of TTD markers in CUE-positive and CUE-negative donations. Our data showed that the prevalence rate of HBV and HCV was significantly higher among CUE-positive donors than among CUE-negative donors. However, due to the low prevalence of HIV (0.1%) and syphilis in the general population in Iran [7] , a difference in risk with CUE status for these markers could not be demonstrated. Our results are consistent with a number of other studies. Brennan et al. showed that CUE was a useful adjunct to routine donor selection and minimized TTDs [8]. Peterson found that CUE-positive donors were 21 times more likely to have HIV Ab, though the impact on blood safety was negligible due to the rarity of window-period donors [4]. Zou et al. showed that CUE was effective for reducing the window period of TTDs, though not as effective as HCV and HIV NAT [9]. Based on an analysis of TTD residual risk, the authors estimated that CUE prevented the collection of 0.2 to 1.3 window-period units. The authors suggested that, before NAT, CUE might have prevented the transfusion of a limited number of infected units[9]. In contrast, in a retrospective and observational study, Cruze found little usefulness of CUE in avoiding major TTDs [10].As NAT is currently unaffordable in Iran, CUE helps to improve blood transfusion safety. The prevalence rates of HBV, HCV and HIV in the general population in Iran have been estimated at 3%, <1% and 0.1%, respectively [7][11]. However, with donor selection programs including CUE and laboratory screening, the corresponding rates in donated blood are 0.41%, 0.12% and 0.004% [12].There are few studies that have evaluated high-risk groups in Iran. HBV, HCV and HIV have similar modes of transmission and are relatively frequent among certain high-risk groups. Vahid found that close contact with an HBV-infected person, extramarital sexual contact and history of sexually transmitted disease were predictors of HBV infection in Iranian blood donors [13]. Alavian et al. showed that intravenous drug abuse is the major risk factor for HCV infection in Iranian blood donors, while sexual promiscuity, defined as one or more extramarital sexual relationships, is also associated with an increased risk of HCV in donors [14]. From these reports, it is apparent that extramarital sexual contact and intravenous drug abuse are important risk factors for HBV, HCV and HIV infection in Iran. Males made up the large majority of donors in our study, and we found a significant association between male gender and CUE positivity. As males may have extramarital activity and unsafe sexual contact, male donors more often have undisclosed risk factors than females.We also found that the majority of first-time donors were CUE positive. This high prevalence suggests that high-risk individuals use donation to find out whether they are infected. Based on the results of this study, as CUE-positive donors might have had higher rates of deferral risk, CUE is a useful tool for improving blood safety in the absence of HCV and HIV NAT.
